# Higher microRNA-221 and lower microRNA-451 expression are associated with poor prognosis in patients with thyroid papillary carcinoma

**DOI:** 10.23938/ASSN.1086

**Published:** 2024-08-23

**Authors:** Jiawei Wu, Weimin Sun, Jingyao Shen, Liping Hu

**Affiliations:** Medical College of Yangzhou University Xuyi Clinical College Department of General Surgery Yangzhou China

**Keywords:** microRNA-451, microRNA-221, Thyroid papillary carcinoma, Pathological features, Prognosis, microRNA-451, microRNA-221, Carcinoma papilar de tiroides, Características patológicas, Pronóstico

## Abstract

**Background:**

To analyze the relationship between serum microRNA-221 and microRNA-451 relative expression and the pathological features and prognosis of patients with thyroid papillary carcinoma.

**Methods:**

Cross-sectional study that included 120 patients with papillary thyroid cancer treated at the hospital and 120 healthy volunteers selected as the control group who underwent physical examination at the same centre. The relative expression levels of microRNA-221 and microRNA-451 were compared between the thyroid papillary carcinoma group (prior to treatment) and the control group. Additionally, microRNA-221 and microRNA-451 expression levels were analyzed in patients with papillary thyroid carcinoma across different pathological characteristics.

**Results:**

Serum microRNA-221 relative levels were significantly higher (p<0.001) in the papillary carcinoma group compared to the control group, while microRNA-451 levels were higher in the control group (p<0.001). In the papillary carcinoma group, microRNA-221 expression was significantly higher in patients with extracapsular invasion (p<0.001), lymphatic metastasis (p=0.003), and poor prognosis (p<0.001). Conversely, microRNA-451 expression was significantly lower (p<0.001) in patients with extracapsular invasion, lymphatic metastasis, and poor prognosis. In the multivariate logistic regression analysis, morphological features suggestive of an aggressive clinical behavior (extracapsular invasion and lymphatic metastasis) were related to high expression of microRNA-221 and low expression of microRNA-451 in patients with thyroid papillary carcinoma (p<0.001).

**Conclusions:**

Serum microRNA-221 and microRNA-451 expression levels are significantly higher and lower, respectively, in patients with papillary thyroid carcinoma, particularly in patients with morphological features suggestive of an aggressive clinical behavior (extracapsular invasion and lymphatic metastasis) and, therefore, of a poor prognosis.

## RESUMEN

**Fundamento:**

Analizar la relación entre la expresión sérica relativa de microRNA-221 y microRNA-451 y las características patológicas y el pronóstico de pacientes con carcinoma papilar de tiroides.

**Metodología:**

Estudio transversal que incluyó 120 pacientes con carcinoma papilar de tiroides tratados en el hospital y 120 voluntarios sanos que se sometieron a un examen físico en el mismo centro (grupo control). Los niveles de expresión relativa de microRNA-221 y microRNA-451 se compararon entre el grupo con carcinoma papilar de tiroides (previo al inicio del tratamiento) y el grupo control, así como entre pacientes con diferentes características patológicas.

**Resultados:**

Los niveles de microRNA-221 estaban más elevados (p<0,001) en el grupo con carcinoma papilar de tiroides, mientras los niveles de microRNA-451 fueron mayores en el grupo control (p<0,001). Los niveles de microRNA-221 eran más altos en pacientes con carcinoma papilar de tiroides con invasión extracapsular (p<0,001), metástasis linfáticas (p=0,003) y mal pronóstico (p<0,001). Los niveles de microRNA-451 fueron significativamente menores (p<0,001) en pacientes con invasión extracapsular, metástasis linfáticas y mal pronóstico. Tanto la invasión extracapsular como las metástasis linfáticas, características sugestivas de un comportamiento clínico agresivo, se asociaron a alta expresión de microRNA-221 y a baja expresión de microRNA-451 en pacientes con carcinoma papilar de tiroides (regresión logística múltiple; p<0,001).

**Conclusión:**

La expresión sérica relativa de microRNA-221 está aumentada y la de microRNA-451 disminuida en pacientes con carcinoma papilar de tiroides, especialmente en aquellos con características patológicas sugestivas de un comportamiento clínico más agresivo y, por tanto, de peor pronóstico (invasión extracapsular y metástasis linfáticas).

## INTRODUCTION

Thyroid cancer is a common malignant tumor with a steadily increasing incidence. Papillary thyroid carcinoma (PTA) is the most common histotype of thyroid cancer^1^. Overall, PTA is characterized by indolent biological behaviors, with clinical symptoms developing over time in most cases. However, some patients may develop aggressive tumors that often invade surrounding tissues such as the cervical trachea, thyroid capsule, cervical esophagus, anterior cervical strap muscles, recurrent laryngeal nerve, and larynx, with early lymph node metastasis and distant metastasis, leading to a relatively high mortality rate^2^^,^^3^. Therefore, actively seeking molecular markers to predict the pathological characteristics and prognosis of PTA is key. Identifying these markers can facilitate early detection of pathological features and outcomes in patients, enabling the timely implementation of targeted and effective treatments.

MicroRNAs (microRNAs) are a class of endogenous non-coding small RNAs typically consisting of 19-23 nucleotides that can negatively regulate gene expression. They can bind to the 3’-untranslated region of target mRNAs, leading to either mRNA degradation or inhibition of protein translation^4^. As important regulators of gene expression, microRNAs play crucial roles in various biological processes, including angiogenesis, cell growth, proliferation, metastasis, and apoptosis. These processes are key to the development and progression of cancer^5^. Studies have demonstrated that microRNAs play significant roles in the occurrence and progression of malignant tumors, including thyroid cancer, breast cancer, and lung cancer. Additionally, microRNAs have the potential to serve as diagnostic markers for certain malignancies^6^^-^^8^.

microRNA-221 is part of the miR-221/222 cluster on the X chromosome and is known for regulating oncogenes and tumor suppressors, including p27^kip1^, which contributes to cancer progression^9^. microRNA-451 is part of the miR-144/451 cluster located on chromosome 17q11.22. It is involved in tumor suppression and regulates the AMPK/mTOR pathway^10^. Both microRNA-221 and microRNA-451 are important members of the microRNA family and are abnormally expressed in patients with thyroid cancer, making them valuable important biological indicators for diagnosing the disease^11^^,^^12^.

microRNA-221 is a primary transcript mainly derived from coding genes and is aberrantly expressed in various tumors. It can regulate tumor metastasis^13^. Previous studies have shown the crucial role of microRNAs in cancer progression, prognosis, and their potential as therapeutic targets. microRNA-221 is known to promote tumor growth and metastasis, while microRNA-451 acts as a tumor suppressor. These regulatory functions underscore the significance of our study. Understanding the expression and impact of microRNA-221 and microRNA-451 in thyroid papillary carcinoma could lead to the development of improved prognostic markers and more effective therapeutic strategies.

Existing studies have primarily focused on the diagnostic value of microRNA-221 and microRNA-451 in thyroid cancer. The goal of this study was to investigate the relationship between serum expressions of microRNA-221 and microRNA-451 and the pathological characteristics and prognosis in PTA patients. Specifically, we aimed to compare the differences in microRNA-221 and microRNA-451 expression levels among PTA patients with varying pathological charac-teristics and prognoses.

## MATERIALS AND METHODS

Cross-sectional study performed in Xuyi Clinical College, Medical College of Yangzhou University, China, from May 2018 to May 2022.

Patients who met the diagnostic criteria for papillary thyroid carcinoma^4^ confirmed by pathological examination and were treated at the hospital during the study period were selected. Inclusion criteria were: patients aged ≥18 years with complete clinical data who had not received chemoradiotherapy or immunotherapy. Exclusion criteria: patients with mental comorbidities, other malignant tumors, circulatory system diseases, diabetes, systemic infection, immune system diseases, thyroid dysfunction, nodular goiter, thyroiditis or thyroid adenoma disease, lactating or pregnant women, infectious diseases, or/and with kidney, liver, heart, and other dysfunctions.

The control group consisted of healthy volunteers who underwent physical examinations at our hospital during the study. Inclusion criteria were: healthy patients with no mental comorbidities or malignant tumors, and complete clinical data.

This study was conducted in accordance with the Declaration of Helsinki and approved by the Xuyi Clinical College, Medical College of Yangzhou University Ethics Committee (LLKSSC2018-10).

Prior receiving treatment, 5 mL of fasting venous blood was collected from each patient. The samples were then centrifuged for 20 minutes using a centrifuge with a centrifugation radius of 10 cm at 1500 rpm. After centrifugations, serum samples were collected and stored at -20 °C until testing.

Next, total RNA was isolated from the serum samples using the TRIzol kit (Thermo Fisher Scientific) according to the manufacturer’s instructions. The concentration and purity of the total RNA were confirmed using a UV-Vis spectrophotometer (Shanghai Aucy Scientific Instruments Co. Ltd, UV1800PC). Two μg of total RNA were taken, 1 μg for reverse transcription using the reverse trans-cription kit (Thermo Fisher Scientific,) to generate cDNA. The reverse transcription product was then amplified by PCR using small nuclear U6 as the internal reference gene (MonScriptRTIIIAll-in-One Mix with dsDNase, Monad) for data normalization. PCR was performed with an initial pre-denaturation at 95º for 5 minutes, followed by 32 cycles with the following conditions: denaturation at 95º for 15 s, restitution at 56º for 20 s, extension at 72º for 45 s. Each sample was run in triplicate. The relative expression levels of microRNA-221 and microRNA-451 were calculated comparing the expression of these microRNAs in PTA patients to those in the healthy control group using the 2^-ΔΔC^ method^10^. The primer sequences of microRNA-221, microRNA-451, and the internal reference are shown in Table 1.

**Table 1:** Sequences of DNA primers used to detect cDNA from microRNAs

	Forward primer sequence	Reverse primer sequence
*microRNA*		
221	5 ‘-CAGCATACATGATTCCTTGTGA-3’	5 ‘-CTTTGGTGTTTGAGATGTTTGG-3’
451	5 ‘-GATCAAGGGGTTTTGGCTTT-3’	5 ‘-GTTGTCCATGGACCTA-3’
*Internal reference*		
U6	5 ‘-GGAGCGAGATCCCTCCAAAAT-3’	5 ‘-GGCTGTTGTCATACTTCTCATGG-3’

The following data were collected:

- Demographic: sex (male, female) and age (<40, ≥40 years).
- Pathological: diameter of the tumor (≤2 cm, >2 cm), extracapsular invasion (yes, no), TNM stage (I-II, III-IV), lymphatic metastasis (yes, no).
- Prognostic: poor prognosis (experiencing recurrence, tumor metastasis, or death after 1 year of follow-up), good prognosis (otherwise).

The outcome measures included the relative expression levels of microRNA-221 and microRNA-451, which were compared between PTA patients and the healthy control group, as well as among PTA patients with different pathological characteristics and prognosis.

SPSS 23.0 software was used for the collation and analysis of the statistical data. The Kolmogorov-Smirnov test was applied to determine whether the sample data conformed to a normal distribution. Quantitative variables were expressed as mean and standard deviation (SD) from the three replicates for each sample and compared using an independent sample t-test. Categorical data were expressed as frequency and percentage and compared using the χ^2^ test. The effects of the variables on microRNA-221 and microRNA-451 expression were analyzed using multivariate logistic regression, reported as *odds ratio* (OR) with their 95% confidence interval (95% CI). A two-tailed p-value <0.05 was considered statistically significantly.

## RESULTS

One hundred and twenty patients with PTA and 120 healthy volunteers as the control group were included in this study.

There were no statistical differences between the PTA and control groups in terms of sex (67.5 vs 69.2% females) and mean age (47.57 years, SD: 3.64, range: 18-82 vs 47.15 years, SD: 3.46, range: 19-84), indicating that the groups were comparable.

The Kolmogorov-Smirnov test confirmed that quantitative variables followed a normal distribution.

The PTA group exhibited higher mean serum microRNA-221 levels (8.50; SD: 1.45 vs 2.49; SD: 0.63; p< 0.001) and lower levels of microRNA-451 (0.87; SD: 0.17 vs 3.23; SD: 0.57; p< 0.001) compared to the healthy control group.

There were no significant differences in the relative expression levels of microRNA-221 and microRNA-451 among PTA patients with different age, sex, tumor diameters, and TNM stages (Table 2). However, patients with morphological features suggestive of aggressive clinical behavior, such as extracapsular invasion and lymphatic metastasis, exhibited higher levels of microRNA-221 and lower levels of microRNA-451. Conversely, PTC patients with a good prognosis showed low expression of microRNA-221 and high expression of microRNA-451 (Table 2).

**Table 2:** Relative expression levels of microRNA-221 and microRNA-451 in patients with papillary thyroid carcinoma across different variables

Variable	n (%)	microRNA-221	p	microRNA-451	p
*Sex*					
Male	39 (32.5)	8.65 (1.51)	0.561	0.88 (0.18)	0.597
Female	81 (67.5)	8.83 (1.62)		0.90 (0.20)	
*Age (years)*					
< 40	78 (65.0)	8.73 (1.57)	0.713	0.85 (0.17)	0.524
≥ 40	42 (35.0)	8.62 (1.53)		0.87 (0.15)	
*Tumor diameter (cm)*					
≤ 2cm	49 (40.8)	8.59 (1.75)	0.52	0.86 (0.19)	0.173
> 2cm	71 (59.2)	8.81 (1.89)		0.82 (0.13)	
*Extracapsular invasion*					
Yes	69 (57.5)	8.93 (1.83)	<0.001	0.79 (0.17)	< 0.001
No	51 (42.5)	7.95 (1.27)		0.92 (0.21)	
*TNM Stage*					
I-II	73 (60.8)	8.32 (1.61)	0.652	0.88 (0.18)	0.344
III-IV	47 (39.2)	8.46 (1.72)		0.85 (0.15)	
*Lymphatic metastasis*					
Yes	42 (35.0)	9.15 (2.15)	0.003	0.65 (0.13)	< 0.001
No	78 (65.0)	8.02 (1.61)		0.94 (0.22)	
*Prognosis*					
Good	87 (72.5)	7.15 (1.05)	< 0.001	1.24 (0.31)	< 0.001
Poor	33 (27.5)	9.72 (2.21)		0.61 (0.15)	

TNM: classification of tumors based on tumor size (T), lymph node affection (N), and presence of metastasis (M).

Extracapsular invasion and lymphatic metastasis continued to be statistically significant in the multivariate logistic regression analysis, identified as independent prognostic factors associated with high relative expression of microRNA-221 and low relative expression of microRNA-451 (Table 3).

**Table 3:** Relationship between clinical and pathological characteristics and the expression levels of microRNA-221 and microRNA-451 in patients with papillary thyroid carcinoma*

Pathological features	microRNA-221	p	microRNA-451	p
	OR (95%CI)		OR (95%CI)	
Extracapsular invasion	2.847	0.001	1.463	< 0.001
	(1.240-22.644)		(1.150-16.865)	
Lymphatic metastasis	3.143	0.003	4.929	< 0.001
	(1.120-8.822)		(1.680-14.458)	

* Final model of logistic multivariate analysis

## DISCUSSION

microRNAs are a class of evolutionarily highly conserved non-coding small RNA molecules, typically 20-24 nucleotides in length. They are transcribed from DNA but are not translated into proteins. microRNAs bind to sequences in the 3’-untranslated region of target genes inhibiting protein translation and silencing these genes either by inducing mRNA degradation or by directly interfering with translation. Approximately 30% of protein-coding genes are regulated by microRNAs with individual microRNAs able to regulate up to 100 target genes. *In vivo*, microRNAs play a significant role in a range biological processes (e.g., differentiation, proliferation, apoptosis, etc.). Previous studies have revealed an association between abnormal non-coding genes and the occurrence and progression of tumors. Since Calin *et al*. first reported the association between microRNA abnormalities and tumors in 2004, increasing evidence has shown that microRNAs play a significant role in the occurrence and progression of malignancies and can even serve as diagnostic markers for certain malignant cancers^11^^,^^12^.

microRNA-221, a primary transcript mainly transcribed from coding genes, is aberrantly expressed in various tumors and participates in the regulation of tumor metastasis^13^. In this study, serum levels of microRNA-221 in the PTA group are higher compared to the healthy control group, with elevated expression observed in patients with lymph node metastasis, extracapsular invasion, and poor prognosis. These results may be attributed to the ability of microRNA-221 to inhibit the expression of relevant target genes, thereby promote tumor occurrence, development, or invasion and metastasis. Previous studies support these findings, showing that microRNA-221 enhances the metastatic potential of cancer cells by downregulating inhibitors of matrix metalloproteinases (MMPs)^14^. Additionally, microRNA-221 inhibits the expression of the cyclin-dependent kinase inhi-bitor p27^kip1^, allowing cancer cells to bypass the G1/S phase arrest and continue proliferating^15^. Moreover, microRNA-221 can also inhibit the expressions of PTEN and TIMP3, promoting cancer cell invasion and metastasis^16^. Consistent with this study´s finding, Yip *et al*.^17^ reported significant correlation between microRNA-221 expression and the invasiveness of thyroid cancer. These findings collectively suggest that microRNA-221 plays a crucial role in the aggressiveness of thyroid papillary carcinoma, underscoring its potential as both a prognostic marker and a therapeutic target.

We show that PTA patients have lower levels of microRNA-451 compared to healthy controls, and those with lymph node metastasis, extracapsular invasion, and poor prognosis exhibit reduced microRNA-451 expression. microRNA-451, located on chromosome 17q11.22, has a completely conserved sequence. It is directly processed into its mature form by AGO, without the need for ribonucleases and is found to be poorly expressed in various malignant tumors^18^. For example, Liu *et al*.^19^^)^ found that microRNA-451 was significantly down-regulated in nasopharyngeal carcinoma, with the extent of down-regulation positively correlated with poor prognosis. Other researchers have reported that microRNA-451 is significantly down-regulated in colorectal cancer, where it inhibits tumor cell growth, proliferation, and invasion by inactivating the PI3K/AKT pathway. Additionally, it can suppress tumor cell proliferation and migration by activating the AMPK pathway^20^^,^^21^. Wang *et al*.^22^ concluded that microRNA-451 regulates cell proliferation by inhibiting the MIF oncogene, a potential target gene of microRNA-451. Therefore, the low expression of microRNA-451 in PTA may promote the occurrence and progression of thyroid cancer by up-regulating the target gene MIF, activating the PI3K/AKT pathway, and inhibiting the AMPK pathway. microRNA-221 and microRNA-451 detection has significant clinical implications. Increased levels of microRNA-221 and decreased levels of microRNA-451 in serum can serve as biomarkers for the early detection and prognosis of thyroid papillary carcinoma. Identifying aggressive pathological features in the patient, allows clinicians to tailor treatment strategies more effectively, potentially improving patient outcomes. These microRNAs may be explored as therapeutic targets, offering new avenues for treatment interventions aimed at modulating their expression to inhibit tumor progression and metastasis.

This study has some limitations. Firstly, with only a few microRNAs types included, it remains unclear whether incorporating additional microRNAs could provide further insight into the pathological characteristics and prognosis of PTA. Secondly, the study excluded patients with other malignant tumors, circulatory system diseases, diabetes, systemic infections, and immune system diseases, which limits its generalizability and applicability. Thirdly, study´s small sample size requires further validation through an expanded cohort. Moreover, we did not investigate the specific genes regulated by microRNA-221 and microRNA-451. Identifying these target genes using bioinformatics tools would provide deeper insight into their roles in thyroid papillary carcinoma. We recommend pursuing this approach in future research.

In conclusion, PTA patients exhibit high serum levels of microRNA-221 and low levels of microRNA-451. Abnormal expression of both microRNA-221 and microRNA-451 in PTA patients is associated with lymphatic metastasis and extracapsular invasion, indicating a strong correlation with poor prognosis. The relative serum level of microRNA-221 and microRNA-451 are valuable for predicting the pathological characteristics and outcomes of PTA, making them potential biomarkers for assessing tumor pathology and prognosis.

## Data Availability

Data are available under reasoned petition to the corresponding author.
